# Novel Hybrid Ligands for Passivating PbS Colloidal Quantum Dots to Enhance the Performance of Solar Cells

**DOI:** 10.1007/s40820-015-0046-4

**Published:** 2015-06-20

**Authors:** Yuehua Yang, Baofeng Zhao, Yuping Gao, Han Liu, Yiyao Tian, Donghuan Qin, Hongbin Wu, Wenbo Huang, Lintao Hou

**Affiliations:** 1 State Key Laboratory of Luminescent Materials & Devices, Institute of Polymer Optoelectronic Materials & Devices, South China University of Technology, Guangzhou, 510640 People’s Republic of China; 2 Siyuan Laboratory, Department of Physics, Jinan University, Guangzhou, 510632 People’s Republic of China

**Keywords:** PbS, Colloidal quantum dot, Solar cells, Ligands

## Abstract

We developed novel hybrid ligands to passivate PbS colloidal quantum dots (CQDs), and two kinds of solar cells based on as-synthesized CQDs were fabricated to verify the passivation effects of the ligands. It was found that the ligands strongly affected the optical and electrical properties of CQDs, and the performances of solar cells were enhanced strongly. The optimized hybrid ligands, oleic amine/octyl-phosphine acid/CdCl_2_ improved power conversion efficiency (PCE) to much higher of 3.72 % for Schottky diode cell and 5.04 % for p–n junction cell. These results may be beneficial to design passivation strategy for low-cost and high-performance CQDs solar cells.

## Introduction

Colloidal quantum dots (CQDs) solar cells as potential next-generation solar energy-harvesting devices have received considerable attention in the past several years [1–4] owing to their low manufacturing cost (coated on substrates using drop-casting, spin-coating or ink-jet printing). Among all kinds of CQDs (such as CdTe [5–9], CdSe [10], PbS [11–14], PbSe [15–17], CuInS_2_ [18], etc.) solar cells, PbS CQDs solar cells have lots of distinctive merits. For example, PbS CQDs solar cells can be prepared in ambient condition under low temperatures below 200 °C, and the electronic bandgap of PbS CQDs can be easily tuned by changing size due to its large exciton Bohr radius (~18 nm for PbS [19]), which enables the fabrication of multi-junction solar cells from single material.

PbS CQDs solar cells with negligible power conversion efficiency (PCE) were first reported in 2005 [20]. There are two key factors which affect the performance of PbS CQDs solar cells. The important one is protection technique of as-prepared PbS CQDs. As the size decreases, the surface state of PbS will go up rapidly due to oxidation if there is no suitable ligand to protect. Devices based on no-protection PbS CQDs exclusively show large internal series resistance and low carrier mobility, resulting in low device performance. Meanwhile, since the long-chain carboxyl acid is usually attached to the surface of PbS, it is difficult to gain satisfactory device performance unless the carboxyl ligands are well removed during device fabrication processes. With development of nanotechnology, many efforts had been made to improve the PCE of PbS CQDs solar cells, including packing and passivation of CQDs [21, 22], adoption of new exchanging ligands [23], and design of new device structure [24]. In order to improve surface passivation and therefore eliminate valence-band-associated trap states in CQDs thin film, Sargent et al. [25–27] first introduced a mixture of CdCl_2_ and tetradecyl phosphonic acid (TDPA) during synthesis process of PbS CQDs. They obtained solar cell device with ~8.0 % efficiency based on these hybrid passivated CQDs. On the other hand, new exchanging ligands such as mercaptopropionic acid (MPA) or di-thiol were introduced during device fabrication process to remove the long-chain carboxyl acid ligands on the surface of PbS CQDs. The thin film prepared by this method was compact and showed good carrier mobility.

In this paper, we developed a novel simple process of passivating PbS CQDs to improve the film quality and therefore to enhance the solar cells’ performance. Different hybrid ligands were introduced during PbS nucleation and QDs growth processes. Two kinds of solar cells based on PbS CQDs were fabricated to verify the effects of ligands passivation.

## Experimental

### Materials

Oleic acid (OA, 90 %), lead oxide (PbO, 99.9 %), 1-octadecene (ODE), CdCl_2_, and oleic amine (OLA) were purchased from Alfa Aesar. Mercaptopropionic acid Hexamethyldisilathiane (TMS), octyl-phosphine acid (OPA), tetradecyl phosphonic acid (TDPA), and octodecyl-phosphine acid (ODPA) were purchased from Aladdin. All chemicals were used directly without any further purification.

### Synthesis of PbS CQDs

PbS CQDs with different passivating ligands were defined as (A) without ligand, (B) CdCl_2_ + OLA + OPA, (C) CdCl_2_ + OLA + TDPA, and (D) CdCl_2_ + OLA + ODPA.

PbS CQDs with different ligands were synthesized by a solvent thermal method reported previously [23]. Typically, 0.45 g PbO, 1.5 mL OA, and 16.5 mL ODE were loaded into a three neck flask at 120 °C and degassed for 6 h to remove any moisture and low boiling point organic solvents. Then, 0.20 mL TMS mixed with 5 mL ODE was quickly injected into the reaction system. The hotplate was removed away immediately, and the reaction was cooled down to room temperature. When the temperature was dropped down to 90 °C, different hybrid ligands *A*, *B*, *C*, or *D* were injected into the reaction system, and the reaction was cool down to room temperature. Then, 50 mL acetone was injected into the final reaction solution to centrifuge at 10,000 rpm for 5 min. Black powder product was collected and re-dissolved into a mixture of 2 mL toluene and 10 mL of ethanol and acetone (volume ratio 1:1) to centrifuge again to remove impurity. This procedure was repeated more than three times to obtain pure PbS CQDs.

The above-mentioned hybrid ligands *B*,* C*, and *D* were prepared by mixing 0.72 mmol CdCl_2_, 2 mL OLA, and respective 0.048 mmol OPA, 0.048 mmol TDPA, and 0.048 mmol ODPA. The mixtures were degassed and refluxed at 90 °C for 5 h until transparent solutions were formed.

### Device Fabrication

**Fig. 1:**
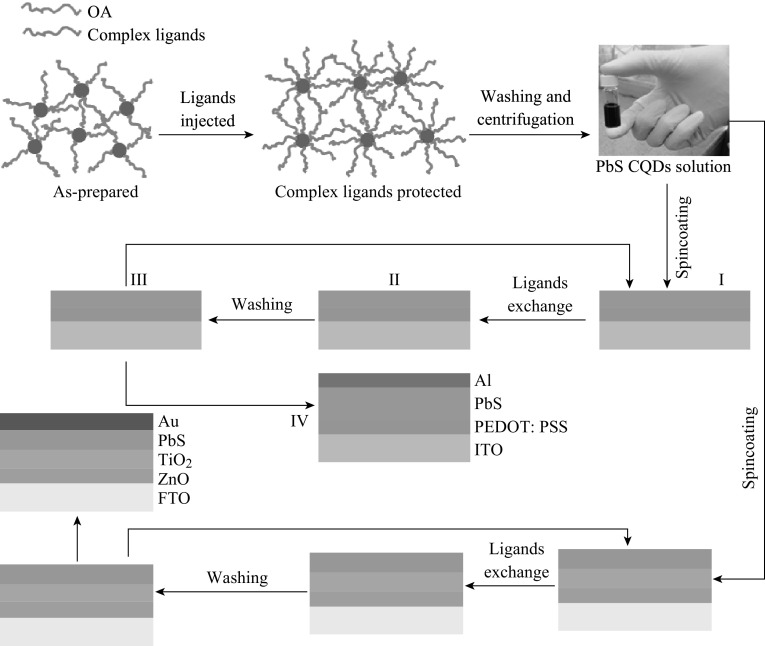
The fabrication schematic of PbS CQDs solar cells passivated by hybrid ligands

Figure 1 illustrates the fabrication processes of PbS CQDs solar cells with Schottky diode structure of ITO/PEDOT:PSS/PbS/Al. In detail, PEDOT:PSS layer was firstly deposited on ITO substrate and baked at 140 °C for 15 h after the substrate was treated with UV-ozone. The PbS CQDs film was deposited using a layer-by-layer spin-coating process under ambient condition as the following steps: (i) PbS CQDs solution was deposited on above PEDOT:PSS layer by spin-coating at 2500 rpm for 15 s; (ii) MPA methanol solution was then spin casted to make ligands exchange; and (iii) Several drops of methanol solvent were deposited and spin casted at 2500 rpm to remove impurities. The procedure from i to iii was repeated for several times until the film thickness reached about 200 nm. Then, the film was baked at 50 °C for 10 h. Finally, Al electrode in thickness of ~80 nm was deposited on the active layer via thermal evaporation through a shadow mask, in which the active area was 0.16 cm^2^.

The fabrication process of solar cells with p–n junction of FTO/ZnO/TiO_2_/PbS/Au was almost similar except that FTO was used as the substrate, and ZnO/TiO_2_ films were inserted between FTO and PbS by thermal decomposition of spin-casting Zn/Ti precursor. The detailed process was described in the literatures [21, 28]. The Au electrode was deposited on the PbS active layer via thermal evaporation.

Space charge limited current (SCLC) measurement was carried out to investigate hole mobility of passivated PbS CQDs thin film. The measurement process was similar to that of PbS CQDs solar cells with Schottky diode structure except that 10 nm MoO_*x*_ and 80 nm Al were deposited on the substrate via evaporation. In this case, the thickness of PbS CQDs thin film was about 200 nm.

### Characterizations

The morphology, structure, and surface state of PbS CQDs were characterized by transmission electron microscope (TEM, JEOL 2010), powder X-ray diffraction (XRD, Bruker D8), and X-ray photoelectron spectroscopy (XPS, Thermo ESCALAB 250), respectively. The optical properties of the samples were recorded by ultraviolet (UV) spectrophotometer (Shimadzu UV-3600). The current–voltage (J–V) curves of solar cells were measured by a source-measurement unit under AM 1.5G spectrum (Keithley 2400) with a solar simulator (Oriel model 91192). The SCLC measurement was carried out on a semiconductor parameter analyzer (Agilent 4155C).

## Results and Discussion

**Fig. 2:**
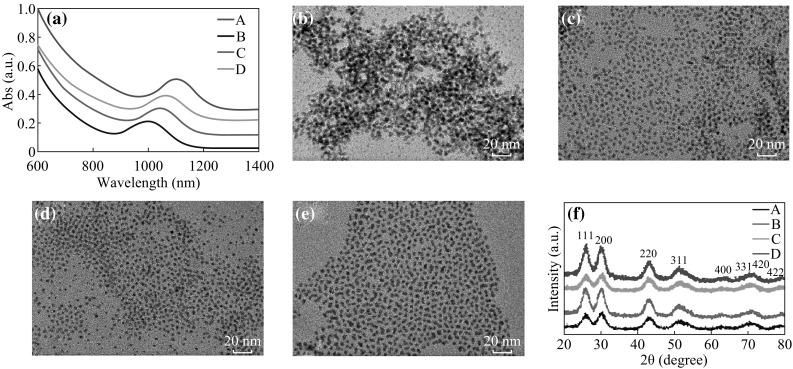
**a** UV absorption spectra of PbS CQDs with different hybrid ligands. TEM images of PbS CQDs with different hybrid ligands: **b** without hybrid ligads (ligand *A*); **c** CdCl_2_ + OLA + OPA (ligand *B*); **d** CdCl_2_ + OLA + TDPA (ligand *C*); **e** CdCl_2_ + OLA +ODPA (ligand *D*). **f** XRD patterns of PbS CQDs thin films with different complex ligands

The UV absorption spectra, TEM images, and XRD patterns of PbS CQDs with different passivating ligands are shown in Fig. 2. The UV absorption peak was at 1102 nm for CQDs without ligand protection (*A*) as shown in Fig. 2a. The peaks blue shifted for those with hybrid ligands and the blue-shift amplitudes increase with increasing carboxyl chain of passivating ligands (998 nm for ligand *B*, 1040 nm for ligand *C*, and 1060 nm for ligand *D*). This may be due to the stronger absorption ability of the alkyl phosphate acid ligands with shorter carboxyl chains which could slow down the growth rate of PbS CQDs. From TEM images shown in Fig. 2b–e, one can see that the CQDs size particles are aggregate and the size without protection is larger of 4.6 nm. The average size for ligand B, C, and D is, 3.5, 3.5 and 3.9 nm, respectively. It was reported that the size decrease of PbS CQDs would result in the blue-shift of absorption peak [4], which is consistent with our results. Figure 2f shows XRD patterns of PbS CQDs with different ligands. The peaks are corresponding to (111), (200), (220), (311), (400), (331), (420), and (420) facets which are in well agreement with the standard cubic structure of PbS (JCPDS 02-0699).

**Fig. 3:**
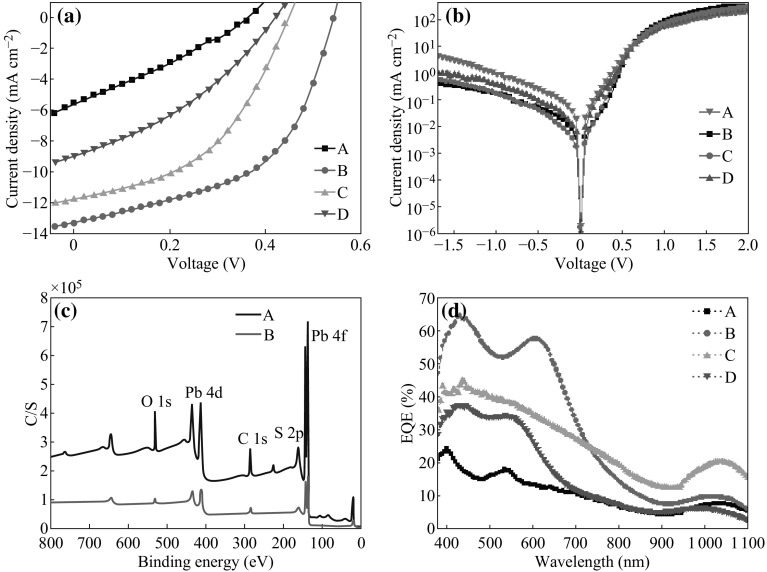
J–V curves of PbS CQDs solar cells with different hybrid ligands **a** under 100 mW cm^−2^ AM1.5 illumination; **b** *under dark*. **c** XPS patterns of PbS thin films (ligands *A* and *B*). **d** EQEs of PbS CQDs solar cells

The J–V curves of CQDs solar cells are shown in Fig. 3a, and the device performances are listed in Table 1. It can be noted that cells with hybrid ligands (*B*, *C*, and *D*) show much higher PCE values than those without ligand (*A*). The cell with ligand B has higher values of *V*_oc_ = 0.54 V, *J*_sc_ = 13.32 mA cm^−2^, and FF = 51.7 %, resulting in PCE = 3.72 %. This value is higher than that of previous reported for PbS CQDs solar cells with the Schottky diode configuration [21, 29, 30]. However, the cell without ligand (*A*) has less values of *V*_oc_ = 0.36 V, *J*_sc_ = 5.541 mA cm^−2^, and FF = 29.07 %, resulting in PCE = 0.58 %. The former is six times higher than the latter. In addition, as the carbon chain length of alky phosphine increases, the values of *J*_sc_, *V*_oc_, and FF decrease accordingly, leading to the decrease of PCE (The PCE is 2.42 % for ligand *C* and 1.32 % for ligand *D*). Figure 3b shows the J–V curves of PbS CQDs solar cells under dark. It is obvious that the dark current of the cell without ligand (*A*) is much higher than those with hybrid ligands (*B*, *C* and *D*). This indicates that cells based on the hybrid ligands efficiently suppress the leakage current at the PbS/Al interface. In the case of no hybrid ligand passivation, PbS CQDs are more likely attacked by oxygen in the reaction system or octane solvent, resulting in the generation of mid-gap trap states. To clarify this, XPS was carried out to characterize the surface state of different PbS thin films as shown in Fig. 3c. The atom percentage of different elements is summarized in Table 2 (Since ligand *C* or *D* has similar results to ligand *B*, their results did not present here). The presence of Cl 2*p* metal chloride peak is related to the CdCl_2_ ligand which was injected during the formation of PbS CQDs in ligand B-passivated PbS. One can note that the peaks of metal oxide and thiol for PbS CQDs without ligand (*A*) appear, whereas no such peaks observed in ligand *B* sample. This indicates that hybrid ligands prevented the oxidation of PbS CQDs during device fabrication process, and MPA was removed thoroughly which was also observed by Sargent et al. [25]. The external quantum efficiency (EQE, see Fig. 3d) of PbS CQDs cells shows better response in the range of 400–800 nm for those with hybrid ligands.

**Table 1:** Photovoltaic parameters of PbS CQDs Schottky diode cell with different hybrid ligands

Devices	*V*_oc_ (V)	*J*_sc_ (mA cm^−2^)	FF (%)	PCE (%)
A	0.36	5.541	29.07	0.58
B	0.54	13.320	51.70	3.72
C	0.44	11.830	46.49	2.42
D	0.42	8.932	35.18	1.32

**Table 2:** Atom percentage of element content of PbS CQDs solar cells with different hybrid ligands from XPS results

Sample	Atom (%)								
	C 1s C–C	C 1s C=O	Cl 2p metal chloride	Pb 4f (4d)	S 2p3 (S 2p1) metal sulfide	S 2p3 (S 2p1) thiol	O 1s carbonates/sulfates	O 1s metal oxide	S 2p3 (S 2p1) sulfate
A-passivated PbS	33.65	5.95	0	10.65	19.01	6.9	20.83	0.57	2.44
B-passivated PbS	36.7	4.94	2.88	11.39	26.81	3.76	13.53	0	0

**Fig. 4:**
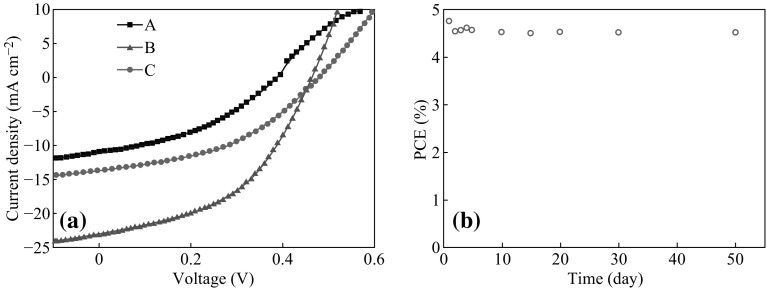
**a** J–V curves of p–n junction PbS CQDs cells with different hybrid ligands (*A*, *B* and *C*). **b** Stability of PbS/TiO_2_ CQDs solar cells devices fabricated using PbS CQDs passivated by ligand B

Figure 4 shows the J–V curves of PbS CQDs p–n junction cells with different hybrid ligands (As devices based on PbS CQDs with ligand *D* have similar results as that with ligand *C*, its J–V curve is not presented here). Their performances are summarized in Table 3. For cell without ligand passivation (A), the PCE was only 1.71 % coupled with low *J*_sc_ = 11.11 mA cm^−2^ and *V*_oc_ = 0.4 V. On the contrary, the cell with ligand *B* passivation shows higher values of *V*_oc_ = 0.46 V, *J*_sc_ = 23.30 mA cm^−2^, and FF = 47 %, resulting in higher PCE = 5.04 %, which is almost three times higher than that of cell without ligand passivation (*A*) and 70 % higher than that of cell with ligand C passivation. It was reported that PbS CQDs solar cells with p-n junction of PbS–TiO_2_ showed good stability over several months [25, 26]. We also tested the stability of the as-prepared cells under ambient conditions in which the cell with ligand B was selected as an example. As shown in Fig. 4b, the device exhibits long-term storage stability in air, and the efficiency decreases less than 5 % after 50 days storage.

**Table 3:** Photovoltaic parameters of PbS CQDs p–n junction cell with different hybrid ligands

Devices	*V*_oc_ (V)	*J*_sc_ (mA cm^−2^)	FF (%)	PCE (%)
A	0.40	11.11	38.5	1.71
B	0.46	23.3	47	5.04
C	0.48	13.81	43.1	2.86

**Fig. 5:**
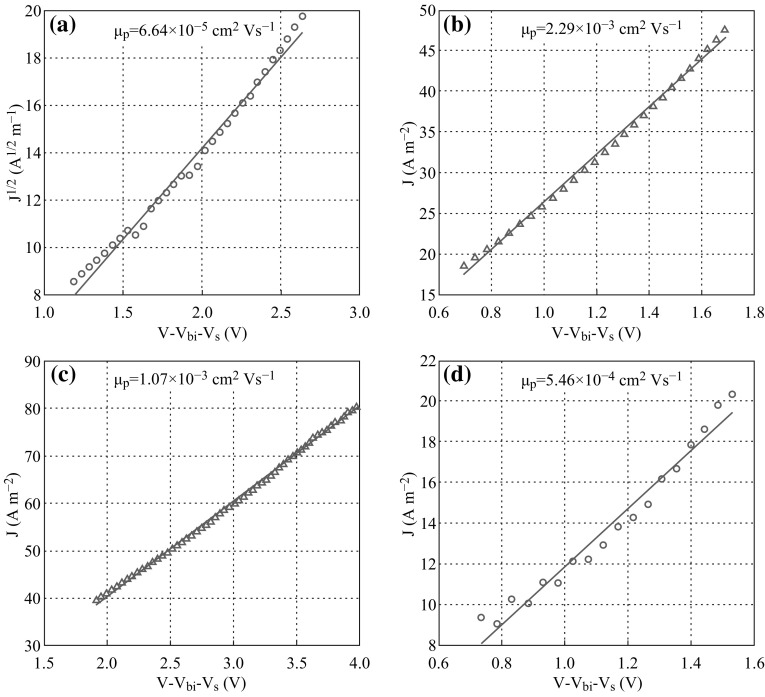
SCLC measurements of PbS CQDs thin films with different hybrid ligands **a** ligand *A*, **b** ligand *B*, **c** ligand *C*, and **d** ligand *D*

Figure 5 shows the SCLC results of PbS CQDs films. The hole mobility was calculated by the formula of $J = \frac{9}{8}\frac{{\varepsilon \mu_{p} V^{2} }}{{L^{3} }},$ [31], where *ε* was the relative dielectric constant, and *L* was the thickness of PbS active layer. A very low hole mobility of 6.64 × 10^−5^ cm^2^ Vs^−1^ was observed in PbS thin film without ligand passivation (*A*), which indicates that large surface states exist in the film. While the hole mobility increases for those with hybrid ligands passivation, and the values are, respectively, 1.07 × 10^−3^ and 5.46 × 10^−4^ cm^2^ Vs^−1^ for ligand *C* and *D*. It reaches the highest of 2.29 × 10^−3^ cm^2^ Vs^−1^ for ligand *B*. The increased hole mobility can improve the cell performance due to recombination reduction of electron and hole during carrier transfer, and therefore, higher *J*_sc_ can be expected. This is consistent with the J–V results (see Table 1). It could be concluded that although CdCl_2_ played a determinative role in mid-gap state passivation [25], the choice of alkyl phosphine acid is more important to improve transport ability associated with the valence band.

## Conclusion

In summary, novel hybrid ligands were developed to passivate PbS CQDs, and the performance of as-prepared PbS CQDs solar cells with not only Schottky diode structure but also p–n junction structure was improved. The reason is that the hybrid ligands passivate surface defects well and prevent oxidation of PbS CQDs during the device fabrication process. In addition, the shorter the chain length of phosphine in hybrid ligands, the higher hole mobility and PCE were demonstrated in cells. Especially, the PbS CQDs cell with ligand B in Schottky diode structure has the highest PCE value compared with reported cells with other ligands. Our results provide an effective way to improve the performance of PbS CQDs solar cells.

## References

[1] Kramer IJ, Levina L, Debnath R, Zhitomirsky D, Sargent EH (2011). Solar cells using quantum funnels. Nano Lett..

[2] Semonin OE, Luther JM, Choi S, Chen HY, Gao J, Nozik AJ, Beard MC (2011). Peak external photocurrent quantum efficiency exceeding 100 % via meg in a quantum dot solar cell. Science.

[3] Gur I, Fromer NA, Geier ML, Alivisatos AP (2005). Air-stable all-inorganic nanocrystal solar cells processed from solution. Science.

[4] Tang J, Sargent EH (2011). Infrared colloidal quantum dots for photovoltaics: fundamentals and recent progress. Adv. Mater..

[5] Panthani MG, Kurley JM, Crisp RW, Dietz TC, Ezzyat T, Luther JM, Talapin DV (2014). High efficiency solution processed sintered CdTe nanocrystal solar cells: the role of interfaces. Nano Lett..

[6] Zhu J, Yang Y, Gao Y, Qin D, Wu H, Hou L, Huang W (2014). Enhancement of open-circuit voltage and the fill factor in CdTe nanocrystal solar cells by using interface materials. Nanotechnology.

[7] Tian Y, Zhang Y, Lin Y, Gao K, Zhang Y (2013). Solution-processed efficient CdTe nanocrystal/CBD-CdS heterojunction solar cells with ZnO interlayer. J. Nanoparticle Res..

[8] Chen Z, Zhang H, Du X, Cheng X, Chen X, Jiang Y, Yang B (2013). From planar-heterojunction to n-i structure: an efficient strategy to improve short-circuit current and power conversion efficiency of aqueous-solution-processed hybrid solar cells. Energy Environ. Sci..

[9] Chen Z, Zhang H, Zeng Q, Wang Y, Xu D, Wang L, Wang H, Yang B (2014). In situ construction of nanoscale CdTe-CdS bulk heterojunctions for inorganic nanocrystal solar cells. Adv. Energy Mater..

[10] Nir Y-G, Michal S-H, Marina Z, Shifi K, Asher S, Nir T (2011). Molecular control of quantum-dot internal electric field and its application to CdSe-based solar cells. Nat. Mater..

[11] Klem EJD, Gregory CW, Cunningham GB, Hall S, Temple DS, Lewis JS (2012). Planar PbS quantum dot/C60 heterojunction photovoltaic devices with 5.2 % power conversion efficiency. Appl. Phys. Lett..

[12] Neo DCJ, Cheng C, Stranks SD, Fairclough SM, Kim JS (2014). Influence of shell thickness and surface passivation on PbS/CdS core/shell colloidal quantum dot solar cells. Chem. Mater..

[13] Kim GH, Walker B, Kim HB, Kim JY, Sargent EH, Park J, Kim JY (2014). Inverted colloidal quantum dot solar cells. Adv. Mater..

[14] Gonfa BA, Zhao H, Li J, Qiu J, Saidani M (2014). Air-processed depleted bulk heterojunction solar cells based on PbS/CdS core-shell quantum dots and TiO_2_ nanorod arrays. Sol. Energy Mater. Sol. C.

[15] Ma W, Swisher SL, Ewers T, Engel J, Ferry VE, Atwater HA, Alivisatos AP (2011). Photovoltaic performance of ultrasmall PbSe quantum dots. ACS Nano.

[16] Tabachnyk M, Ehrler B, Gelinas S, Bohm ML, Walker BJ (2014). Resonant energy transfer of triplet excitons from pentacene to PbSe nanocrystals. Nat. Mater..

[17] Gao J, Luther JM, Semonin OE, Ellingson RJ, Nozik AJ, Beard MC (2011). Quantum dot size dependent J-V characteristics in heterojunction ZnO/PbS quantum dot solar cells. Nano Lett..

[18] Weil BD, Connor ST, Cui Y (2010). CuInS_2_ solar cells by air-stable ink rolling. JACS.

[19] Hines MA, Scholes GD (2003). Colloidal PbS nanocrystals with size-tunable near-infrared emission: observation of post-synthesis self-narrowing of the particle size distribution. Adv. Mater..

[20] McDonald SA, Konstantatos G, Zhang S, Cyr PW, Klem EJD, Levina L, Sargent EH (2005). Solution-processed PbS quantum dot infrared photodetectors and photovoltaics. Nat. Mater..

[21] Tang J, Wang X, Brzozowski L, Barkhouse DAR, Debnath R, Levina L, Sargent EH (2010). Schottky quantum dot solar cells stable in air under solar illumination. Adv. Mater..

[22] Luther JM, Law M, Beard MC, Song Q, Reese MO, Ellingson RJ, Nozik AJ (2008). Schottky solar cells based on colloidal nanocrystal films. Nano Lett..

[23] Zhitomirsky D, Furukawa M, Tang J, Stadler P, Hoogland S, Voznyy O, Liu H, Sargent EH (2012). N-type colloidal-quantum-dot solids for photovoltaics. Adv. Mater..

[24] Liu H, Tang J, Kramer IJ, Debnath R, Koleilat GI (2011). Electron acceptor materials engineering in colloidal quantum dot solar cells. Adv. Mater..

[25] Ip AH, Thon SM, Hoogland S, Voznyy O, Zhitomirsky D, Debnath R, Levina L, Amassian A, Sargent EH (2012). Hybrid passivated colloidal quantum dot solids. Nat. Nanotechnol..

[26] Chuang CM, Brown PR, Bulovic V, Bawendi MG (2014). Improved performance and stability in quantum dot solar cells through band alignment engineering. Nat. Mater..

[27] Ning Z, Voznyy O, Pan J, Hoogland S, Adinolfi V (2014). Air-stable n-type colloidal quantum dot solids. Nat. Mater..

[28] Kramer LJ, Sargent EH (2014). The architecture of colloidal quantum dot solar cells: materials to devices. Chem. Rev..

[29] Yoon W, Boercker JE, Lumb MP, Placencia D, Foos EE, Tischler JG (2013). Enhanced open-circuit voltage of PbS nanocrystal quantum dot solar cells. Sci. Rep.-UK.

[30] Malgras V, Nattestad A, Yamauchi Y, Dou SX, Kim JH (2015). The effect of surface passivation on the structure of sulphur-rich PbS colloidal quantum dots for photovoltaic application. Nanoscale.

[31] Ciach R, Dotsenko YP, Naumov VV, Shmyryeva AN (2003). Injection technique for the study of solar cell test structures. Sol. Energy Mater. Sol. Cells.

