# Erratum to: Novel Hybrid Ligands for Passivating PbS Colloidal Quantum Dots to Enhance the Performance of Solar Cells

**DOI:** 10.1007/s40820-015-0078-9

**Published:** 2016-01-16

**Authors:** Yuehua Yang, Baofeng Zhao, Yuping Gao, Han Liu, Yiyao Tian, Donghuan Qin, Hongbin Wu, Wenbo Huang, Lintao Hou

**Affiliations:** 1 State Key Laboratory of Luminescent Materials & Devices, Institute of Polymer Optoelectronic Materials & Devices, South China University of Technology, Guangzhou, 510640 People’s Republic of China; 2 Siyuan Laboratory, Department of Physics, Jinan University, Guangzhou, 510632 People’s Republic of China

## Erratum to: Nano-Micro Lett. (2015) 7(4):325–331 DOI 10.1007/s40820-015-0046-4

In the original publication, the accepted date was published incorrectly. The correct accepted date should read as follows: “Accepted: 30 May 2015”

